# High-amylose barley bread improves postprandial glycemia compared to regular barley and wheat bread in subjects with or without type 2 diabetes

**DOI:** 10.1038/s41430-025-01646-6

**Published:** 2025-07-21

**Authors:** Mette Bohl, Søren Gregersen, Zhihang Li, Andreas Blennow, Kim H. Hebelstrup, Kjeld Hermansen

**Affiliations:** 1 Steno Diabetes Center Aarhus, Aarhus University Hospital, 8200 Aarhus N, Aarhus, Denmark; 2 Medical Diagnostic Center, University Clinic for Innovative Patient Pathways, Regional Hospital Central Jutland, Silkeborg, Denmark; 3 Department of Clinical Medicine, Aarhus University, 8200 Aarhus N, Aarhus, Denmark; 4 Department of Plant and Environmental Science, University of Copenhagen, 1871 Frederiksberg C, Denmark; 5 Blennow Holding AB, Malmö, Sweden; 6 Department of Agroecology, Section for Crop Genetics and Biotechnology, Aarhus University, 4200 Slagelse, Denmark; 7 Plantcarb Aps, 2970 Hørsholm, Denmark

**Keywords:** Type 2 diabetes, Nutrition

## Abstract

**Background:**

Genetically modified (GMO) high-amylose barley lowers postprandial glucose. Since certain EU countries do not allow GMO barley, we therefore assessed if high-amylose barley made from traditional breeding (Lean Baking Barley, LBB) lowers postprandial glucose compared to bread made from regular barley (RB) or wheat (WF) in individuals with or without type 2 diabetes (T2D).

**Methods:**

In a randomised crossover design, 38 participants (18 T2D and 20 non-T2D) consumed 160 g of bread made from 100% LBB, RB, or WF. Postprandial metabolic responses, appetite and bread perception were measured. A mixed model ANOVA was used for analysis.

**Results:**

LBB bread reduced 4 h postprandial glucose measured as incremental area under the curve (iAUC) by 41% and 39% vs. WF and RB bread in T2D and by 28% and 32% in non-T2D (all, *P* < 0.05). In T2D, LBB reduced postprandial insulin (iAUC) by 52% and 38% vs. WF and RB, and by 60% vs. WF in non-T2D (all, *P* < 0.05). Postprandial GIP (iAUC) was lower after LBB in both groups vs. RB and WF (*P* < 0.05). GLP-1 (iAUC) and FFA (tAUC) were lower after LBB vs. WF in non-T2D (*P* < 0.05), but not in T2D. Appetite scores were similar for all breads. Overall liking was higher for WF but did not differ between barley types.

**Conclusion:**

LBB breads reduce postprandial glucose and insulin compared to RB and WF bread in individuals irrespective of T2D. LBB may have potential as a functional food in prevention and management of T2D.

ClinicalTrails.gov registration: NCT04702672.

## Introduction

Diabetes management, including the prevention and remission of type 2 diabetes (T2D), relies on effective evidence-based studies. Well-designed dietary recommendations and nutrition therapy are essential to improve both life expectancy and quality [1, 2]. Among the key factors implicated in the development and progression of diabetes is increased postprandial glucose levels following the consumption of high-glycemic index foods, e.g. wheat flour bread [3]. Dietary strategies aimed at modulating postprandial glucose responses have therefore emerged as promising interventions to mitigate the risk and progression of T2D.

Both high-amylose flour and barley flour have garnered considerable attention in this context due to their unique nutritional properties. High-amylose flour, characterised by a higher proportion of resistant starch, has been shown to elicit a blunted postprandial glucose response compared to traditional flours high in rapidly digestible starches [4, 5]. Similarly, barley flour, rich in dietary fibre, especially beta-glucans, exhibits a slower and more sustained release of glucose into the bloodstream, thus exerting favourable effects on glycemic control and appetite [6–8].

We have previously demonstrated that by replacing 50% of wheat flour with 50% of genetically modified high-amylose barley flour [9] postprandial glucose responses were reduced by 34% compared with 100% wheat flour bread [10]. However, genetically modified flour is not a commercially viable option in many European countries. Consequently, we have developed a high-amylose barley variety, termed Lean Baking Barley (LBB), using publicly accepted mutation-based breeding protocols [11].

The present study aims to investigate the postprandial glycemic responses of bread baked with LBB flour compared to bread baked with either regular barley flour (RB) or wheat flour (WF). Additionally, we measured acute postprandial changes in satiety, incretins, lipids and glucagon. Furthermore, evaluation of test breads in regard to look, taste, texture, and overall liking were performed.

We hypothesised that bread made from LBB has the potential to improve postprandial glucose responses, measured as incremental area under the curve (iAUC), compared to both RB and WF bread in subjects with or without T2D, respectively.

## Methods

### Study design

This study was performed as an acute, single blind, randomised, controlled, crossover trial with three test meals consisting of bread made with either 100% LBB, 100% RB, or 100% WF. Randomisation was done using RedCap®.

The primary outcome was changes in postprandial glucose (given as 4 h iAUC) in subjects with or without T2D. The secondary outcomes were postprandial changes in insulin, glucagon, triglyceride (TG), FFA, gastric inhibitory polypeptide (GIP) and glucagon-like peptide-1 (GLP-1) calculated as either iAUC or total AUC (tAUC). Furthermore, questionnaires were completed to evaluate e.g. satiety and fullness, as well as perception scores of the test meals.

The study was registered at ClinicalTrails.gov as NCT04702672.

### Participants

Adults with and without T2D were recruited via local newspapers and online ads. The study took place at Steno Diabetes Centre Aarhus, Aarhus University Hospital, between February and June 2024. All participants provided written informed consent after receiving oral and written information. Eligibility was determined through physical exams, medical history, and blood tests.

Inclusion criteria without T2D: Adults ( ≥ 18 years) without any form of diabetes. With T2D: Adults ( ≥ 18 years) diagnosed according to IDF criteria, with HbA1c between 6 and 9.3% (42–78 mmol/mol).

Exclusion criteria (both groups): Insulin use, once-weekly GLP-1 agonists, acarbose, significant cardiovascular, kidney, liver, psychiatric, or endocrine conditions, steroid treatment, substance abuse, pregnancy, breastfeeding, or legal incompetence. Stable treatment for hypertension or high cholesterol was permitted.

### Experimental protocol

After a standardised evening meal and an overnight fast (from midnight), the study participants arrived at the clinic at 07.30 AM on all three study days.

Smoking was not allowed during the overnight fast or the study visits. Alcohol consumption was not permitted the day before the study days. Anti-hypertensive, cholesterol-lowering and anti-diabetic drugs were paused 24 h before every study day. The three intervention days were separated by a six-day minimum washout.

Antihypertensive medications were temporarily discontinued due to their potential confounding effects on blood glucose regulation, although blood pressure was not an outcome measure in this study. The half-lives of metformin and simvastatin - used by the majority of study participants - are approximately 2–4 h, while those of SGLT-2 inhibitors, DPP-4 inhibitors, and atorvastatin range from 12 to 13 h. We acknowledge that a one-day discontinuation may not allow for complete washout of all drugs across there therapeutic classes; however, due to practical considerations, all medications were paused for the same duration.

During the study days, a catheter was placed in a cubital vein for blood sampling. Baseline questionnaires were completed, and blood samples were drawn. At 0 min the test bread was consumed within the next 10 min along with 250 ml of tap water. A bread perception questionnaire was completed within the first 10 min.

During the following 4 h, blood samples were drawn as following: glucose, insulin, and glucagon at –10, 0, 10, 20, 30, 45, 60, 90, 120, 150, 180, 210, and 240 min; TG, FFA, GLP-1 and GIP at –10, 0, 30, 60, 120, 180, and 240 min. All blood samples were immediately centrifuged at 3000 g for 10 min at 4 °C; thereafter, plasma samples were frozen at –20 °C and the next day stored at –80 °C, except from plasma for glucose measurements, since this was analysed on the study day.

Questionnaires regarding satiety were completed at: 0, 30, 60, 120, 180, and 240 min. At 120 min, an additional 250 ml of tap-water was served.

### Study breads

Regular nude barley (RB, *H. vulgare* var. *nudum* PS3) and high-amylose nude barley (LBB, *H. vulgare* var. *nudum* LBB) were grown in 2023 at Aarhus University, Flakkebjerg. RB was developed by Agrologica (Mariager, Denmark). Grains from both varieties were milled using a Komo Fidibus 21 (KOMO GmbH, Germany). The LBB variety was bred to lack Starch Branching Enzyme IIa (SBEIIa), resulting in 46.5% amylose content. Its yield, grain weight, and starch granule shape were similar to the original line, as described in detail previously [12]. Wheat bread was made with commercial Manitoba flour (HavneMøllen, Denmark). All three bread types followed similar recipes and were produced by P.A. Andersen Bakery (Vejle, Denmark). Breads were portioned (160 g), sealed, frozen at −20 °C, defrosted overnight before study days, and served unheated.

Participants consumed a standard commercial spaghetti bolognese meal (1750 kJ; 15.4 g fat, 49 g carbs, 18.2 g protein) the night before each study day (Salling Group A/S, Denmark). Extra foods were allowed if intake was measured and replicated across all test days.

### Bread component analysis

Bread analysis were performed on breads prepared similarly to the study breads.

The moisture content was determined by the weight loss after drying in a vacufuge vacuum concentrator from Eppendorf overnight. The total carbohydrate content was measured as the sum of the dietary fibre and starch content. The Megazyme total starch assay kit (K-TSTA-100A, Wicklow, Ireland) was used to determine the total starch content of samples containing resistant starch following the manufacturer’s instructions. This method variant uses dimethyl sulfoxide and a boiling bath, and dissolution in dimethyl sulfoxide at 100 °C is effective for solubilizing all starches in the bread. The dietary fibre content was determined using the Megazyme total fibre assay kit (K-TDFR-200A, Wicklow, Ireland) in accordance with the manufacturer’s instructions. This method variant uses 1 h incubation with heat-stable α-amylase, which is a critical enzymatic digestion step to remove digestible starch components. The resistant starch content was determined using the Megazyme resistant starch assay kit (K-RAPRS, Ireland).

### Blood analyses

Plasma glucose was measured by enzyme sensor technology Xylem Brand on YSI 2500 or 2900 (YSI Incorporated, Ohio, USA). EDTA-plasma insulin and glucagon were measured with ELISA (insulin no. 10- 1113-01 and glucagon no. 10-1271-01; Mercodia AB, Sweden). Plasma FFA concentrations were measured with enzymatic colorimetric assays by using commercial kits (code 270–7700, Wako Chemicals GmbH, Germany) on the NOVI apparatus (Perkin Elmer, Connecticut, USA). Triglycerides were measured on an Indiko apparatus with quantitative enzymatic methods using commercial kits (REF 981786, Thermo Fisher Scientific, Roskilde, Denmark). GLP-1 and GIP were measured with NL-ELISA techniques (GLP-1 no. 10-1278-01 and GIP no. 10-1258-01; Mercodia AB, Sweden) on the Multimode Plate Reader EnVision (Perkin Elmer, Connecticut, USA).

### Questionnaires

When consuming the test meal (time 0–10 min), the participants evaluated the looks, texture, taste, and overall liking of the bread. The evaluation consisted of seven boxes rating from the most negative “1; do not like”, over “3; neither/nor” to the most positive “7; like very much”.

Visual analogue scale (VAS) was used to assess hunger, satiety, fullness, desire to eat and prospective consumption of the test breads. VAS consists of a 150 mm line scale with words anchored at each end, expressing the most negative and the most positive rating. The questionnaires were made on paper at time 0, 30, 60, 120, 180 and 240 min a new paper was used for every time point [13, 14]. Results were converted from mm to percentage when results were analysed.

### Statistical analysis

The power calculation was made to detect a difference in our primary outcome (i.e., postprandial glucose response, given as iAUC) of 20% between diets [10]. The number of participants needed to complete the study and achieve a statistical power of 80% was calculated to be 18 subjects with T2D and 20 subjects without T2D (a < 0.05, b = 0.80). A mixed model ANOVA was used to examine the difference between bread types. *P* < 0.05 was considered statistically significant. Results are given as mean ± 95% confidence interval (CI) in tables and as mean ± SEM in graphs, unless otherwise stated. All statistical calculations were performed with STATA version 18 (StataCorp LP, Texas, USA) and graphical elements were generated using GraphPad Prism 10 (GraphPad Software, Boston, USA).

## Results

### Baseline clinical characteristics

Thirty-eight participants were randomised and 36 completed the study. The two dropouts were due to personal reasons; there was one dropout in each group.

Table 1 presents baseline characteristics of the 36 completing participants. The group with T2D consisted of significantly less women, had higher HbA1c, lower cholesterols and blood pressure, and more subjects were treated with statins and antihypertensive drugs than the non-T2D group. These findings are expected due to international guidelines for diabetes care striving towards lower levels of lipids and blood pressure in subjects with, than without, T2D.

**Table 1:** Baseline characteristics of the 36 completing subjects*.

	T2D group (*n* = 17)	Non-T2D group (*n* = 19)	*P*-value T2D vs. non-T2D
Female sex, *n* (%)	3 (17.7%)	11 (55.0%)	0.029
Age, *y*	71.9 (69.5, 74.3)	64.9 (57.6, 72.2)	0.064
T2D, *n* (%)	17 (100%)	0 (0%)	–
Weight, *kg*	85.7 (80.4, 91.0)	78.1 (71.0, 85.2)	0.145
BMI (kg/m^2^)	27.3 (25.8, 28.9)	25.8 (23.7, 28.0)	0.351
Smoking, *n*	1 (6%)	0 (0%)	0.297
Haemoglobin A1c (%)	7.0 (6.5, 7.5)	5.4 (5.3, 5.6)	<0.001
Haemoglobin A1c (mmol/mol)	54 (48, 59)	36 (34, 38)	<0.001
Total cholesterol (mmol/L)	4.0 (3.5, 4.4)	5.0 (4.5, 5.5)	0.005
LDL cholesterol (mmol/L)	1.9 (1.5, 2.3)	2.9 (2.5, 3.3)	0.001
HDL cholesterol (mmol/L)	1.3 (1.1, 1.4)	1.6 (1.4, 1.8)	0.014
Systolic blood pressure (mmHg)	134 (126, 142)	142 (133, 151)	0.226
Diastolic blood pressure (mmHg)	78 (74, 82)	83 (80, 87)	0.039
Statin use, *n*	14 (82%)	6 (30%)	<0.001
Drug treatment for hypertension, *n*	11 (65%)	4 (20%)	0.007
Metformin use, *n*	16 (94%)	0 (0%)	–
SGLT-2 inhibitor use, *n*	3 (17%)	0 (0%)	–
DPP4-inhibitor use, *n*	3 (17%)	0 (0%)	–

^*^Values are means, 95% CI in parentheses unless otherwise stated. T2D; type 2 diabetes. SGLT-2 inhibitor; sodium-glucose cotransporter-2 inhibitor. DPP4-inhibitor; dipeptidyl peptidase-4 inhibitor.

### Bread assessment

Table 2 presents the moisture content, total carbohydrate, total fibre, total starch and resistant starch content in g per 100 g of each bread sample. Total carbohydrate content is the sum of total starch and dietary fibre.

**Table 2:** The moisture, total carbohydrate, total fibre, total starch and resistant starch content of bread samples*.

	Moisture content (g/100 g)	Total carbohydrate (g/100 g)†	Dietary fibre content (g/100 g)	Total starch content (g/100 g)	Resistant starch content (g/100 g)
WF	40.0 ± 1.0^b^	45.9 ± 0.9^d^	2.5 ± 0.2^e^	43.4 ± 0.8^e^	0.8 ± 0.1^d^
RB	40.2 ± 0.6^b^	49.7 ± 1.9^c^	4.4 ± 0.2^d^	45.3 ± 1.7^d^	0.9 ± 0.1^c^
LBB	42.0 ± 2.0^a^	50.3 ± 0.8^c^	6.6 ± 0.3^b^	43.8 ± 0.7^e^	1.1 ± 0.1^b^

^*^Values are mean values ± SD. Values with different letters (a,b,c,d,e) in the same column are significantly different at *P* < 0.05. †Total carbohydrate is the sum of dietary fibre and total starch. *WF* wheat flour, *RB* regular barley flour, *LBB* lean baking barley flour (high-amylose barley flour).

Both barley bread contained more dietary fibre and resistant starch than WF, with LBB containing even more than RB. The amount of carbohydrate did not differ between LBB and RB but was higher than in the WF bread.

### Glucose, insulin and glucagon

**Fig. 1:**
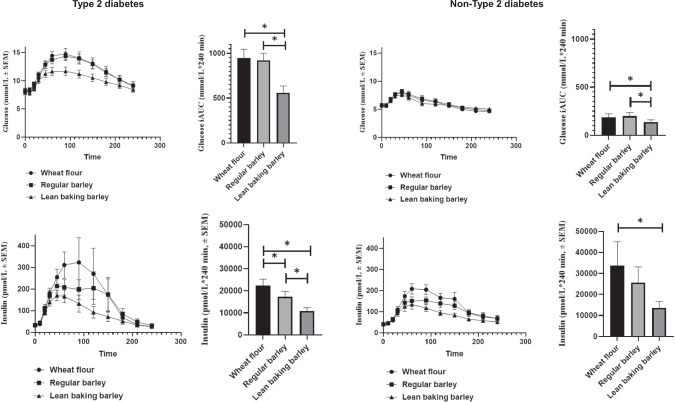
In the top row, changes in glucose are shown alongside the corresponding incremental area under the curve (iAUC). To the left, curves represent the 17 completing participants with type 2 diabetes (T2D) in response to test meals of either 100% wheat flour bread (WF), 100% regular barley flour bread (RB), or 100% high-amylose barley (Lean Baking Barley, LBB). To the right, curves show postprandial glucose responses in the 19 completing participants without T2D. The bottom row displays changes in insulin alongside the corresponding iAUC for the T2D group (left) and the group without T2D (right). *Significantly different from each other (*P* < 0.05). SEMS are indicated.

Fasting concentrations of glucose, insulin and glucagon are shown in Table 3, alongside 4 h postprandial responses such as iAUC (glucose and insulin) or tAUC (glucagon) for the T2D and the non-T2D group. The postprandial changes for glucose and insulin as well as their corresponding iAUC for the T2D and the non-T2D group (Fig. 1) demonstrated that, in the T2D group, postprandial glucose was reduced by 41% (*P* < 0.001) after LBB compared to WF and was reduced by 39% (*P* < 0.001) compared to RB.

**Table 3:** Fasting and postprandial plasma glucose, insulin, glucagon, lipids and incretins*.

	100% WF (T2D)	100% RB (T2D)	100% LBB (T2D)	100% WF (non-T2D)	100% RB (non-T2D)	100% LBB (non-T2D)
Glucose fasting (mmol/l)	8.2 (7.3, 9.1)	8.2 (7.3, 9.1)	7.9 (7.0, 8.8)	5.7 (5.3, 6.1)	5.7 (5.4, 6.1)	5.7 (5.3, 6.1)
Glucose iAUC (mmol/l*240 min)	948 (781, 1115)^a^	923 (756, 1090)^b^	559 (392, 726)^a,b^	188 (122, 253)^x^	201 (136, 266)^y^	136 (71, 201)^x,y^
Insulin fasting (pmol/l)	39.2 (29.3, 49.1)	40.5 (30.6, 50.4)	41.5 (31.7, 51.4)	31.6 (21.2, 42.0)	33.7 (23.3, 44.0)	33.1 (22.8, 43.5)
Insulin iAUC (nmol/l*240 min)	22.5 (17.8, 27.2)^a,b^	17.3 (12.6, 21.9)^a,c^	10.8 (6.1, 15.4)^b,c^	33.9 (18.1, 49.6)^x^	25.7 (9.9, 41.4)	13.7 (−2.1, 29.4)^x^
Glucagon fasting (pg/ml)	6.4 (5.1, 7.8)	7.2 (5.9, 8.6)	7.1 (5.8, 8.5)	5.2 (4.2, 6.2)	5.5 (4.5, 6.5)^x^	4.6 (3.6, 5.6)^x^
Glucagon tAUC (pg/ml*240 min)	606 (468, 745)	616 (477, 754)	550 (411, 688)	383 (266, 501)	495 (378, 613)^y^	286 (169, 404)^y^
TG fasting (mmol/l)	1.46 (1.07, 1.85)	1.50 (1.11, 1.89)	1.50 (1.11, 1.89)	1.13 (0.96, 1.29)	1.07 (0.91, 1.24)	1.03 (0.87, 1.19)
TG tAUC (mmol/l*240 min)	340 (247, 432)	342 (250, 435)	346 (253, 438)	257 (215, 299)	242 (201, 284)	235 (193, 277)
FFA fasting (mmol/l)	0.64 (0.57, 0.71)	0.67 (0.6, 0.74)	0.70 (0.60, 0.79)	0.60 (0.47, 0.73)	0.59 (0.47, 0.72)	0.55 (0.49, 0.67)
FFA tAUC (mmol/l*240 min)	88.1 (72.5, 103.6)	92.6 (77.0, 108.2)	83.9 (68.3, 99.4)	90.8 (69.0, 112.6)^x^	88.0 (66.2, 109.8)	70.1 (48.3, 91.9)^x^
GIP fasting (pmol/l)	7.83 (6.18, 9.48)	9.43 (6.65, 12.20)	9.16 (6.83, 11.49)	7.48 (5.91, 9.05)	7.06 (5.78, 8.34)	7.27 (5.92, 8.62)
GIP iAUC (nmol/l*240 min)	7.4 (6.1, 8.7)^a,b^	4.9 (3.6, 6.2)^a,c^	2.6 (1.3, 3.9)^b,c^	9.6 (7.7, 11.5)^x,y^	6.7 (4.8, 8.6)^x,z^	4.5 (2.6, 6.5)^y,z^
GLP-1 fasting (pmol/l)	5.03 (3.89, 6.16)	4.74 (4.03, 5.45)	4.96 (4.22, 5.70)	3.61 (2.97, 4.27)	3.80 (3.22, 4.39)	3.61 (3.00, 4.23)
GLP-1 iAUC (pmol/l*240 min)	503 (326, 679)	474 (298, 651)	447 (270, 623)	790 (580, 999)^x^	623 (415, 833)	442 (233, 651)^x^

^*^Values are means values ± 95% CI in parentheses. Values with similar subscript letters in same row, divided by group with type 2 diabetes (T2D) (a,b,c) or without T2D (non-T2D) (x,y,z), differ from each other (*P* < 0.05). *FFA* free fatty acids, *GIP* gastric inhibitory polypeptide, *GLP-1* glucagon like peptide, *iAUC* incremental area under curve, *LBB* lean baking barley (high-amylose barley), *RB* regular barley, *tAUC* total area under curve, *TG* triacylglycerol, *WF* wheat flour.

In the non-T2D group, reductions in postprandial glucose for LBB were 28% (*P* = 0.009) and 32% (*P* = 0.001) compared to WF and RB, respectively.

Surprisingly no significant differences in postprandial glucose responses (iAUC) were observed between RB and WF in the T2D nor in the non-T2D group.

In the T2D group, postprandial insulin responses (iAUC) for LBB were reduced by 52% (*P* < 0.001) and 38% (*P* < 0.001) compared to WF and RB, respectively. RB reduced insulin responses by 23% (*P* = 0.001) compared to WF in the T2D group.

In the non-T2D group, postprandial insulin responses (iAUC) for LBB were reduced by 60% (*P* = 0.002) compared to WF. There was a trend towards lower postprandial insulin after LBB compared to RB, however, not significant (*P* = 0.053).

In the T2D group, postprandial glucagon responses did not differ between groups (*P* > 0.05).

In the non-T2D group, fasting glucagon levels were lower on the LBB intervention day compared to the RB intervention day. In the non-T2D population postprandial glucagon responses (tAUC) were reduced after RB compared to LBB (*P* = 0.002).

### FFA and TG

Fasting and 4 h postprandial changes (tAUC) in plasma concentrations of FFA and TG are shown in Table 3.

In the T2D group, postprandial FFA responses (tAUC) did not differ between groups. In the non-T2D group, postprandial FFA (tAUC) was reduced by 23% (*P* = 0.027) after LBB compared to WF, but did not significantly differ from RB (*P* = 0.055).

Postprandial TG (tAUC) did not differ between interventions regardless of T2D status.

### GIP and GLP-1

Fasting and 4 h postprandial changes (iAUC) in GIP and GLP-1 are shown in Table 3.

In the T2D group, postprandial GIP responses (iAUC) for LBB were reduced by 65% (*P* < 0.001) and 47% (*P* < 0.001) compared to WF and RB, respectively. GIP was reduced after RB by 33% (*P* = 0.001) compared to WF.

In the non-T2D group the same pattern was found. Postprandial GIP after LBB was reduced by 52% (*P* < 0.001) and 32% (*P* = 0.012) compared with after WF and RB, respectively. Postprandial GIP response after RB was reduced by 30% (*P* = 0.002) compared to WF.

In the T2D group, no differences were found between interventions on postprandial GLP-1 responses.

In the non-T2D, postprandial GLP-1 was reduced after LBB compared to WB (*P* = 0.004 and *P* = 0.006, respectively). In the non-T2D group no differences were seen between LBB and RB, nor between RB and WF, regarding postprandial GLP-1.

### Bread perceptions and satiety

The participants’ evaluation of the looks, texture, taste and overall liking of the different breads showed that the WF bread performed better than both LBB and RB on all parameters (*P* < 0.05). In terms of appearance, RB bread scored better than LBB bread; however, on the other parameters, we found no differences between the barley breads (Table 4).

**Table 4:** Bread perception analyses, ranked from 1= ’do not like’ to 7= ’like very much’*.

Bread types	Looks	Texture	Taste	Overall
Wheat (WF)	5.8 (5.4, 6.1)^a^	5.4 (5.9, 5.7)^a^	5.4 (4.9, 5.8)^a^	5.4 (5.0, 5.8)^a^
Regular barley (RB)	3.9 (3.4, 4.4)^b^	2.7 (2.2, 3.2)^b^	2.8 (2.3, 3.4)^b^	2.7 (2.2, 3.1)^b^
Lean baking barley (LBB)	3.2 (2.7, 3.7)^c^	2.5 (2.2, 2.8)^b^	2.4 (2.0, 2.7)^b^	2.4 (2.1, 2.8)^b^

^*^ Values are mean values ± 95% CI in parentheses. Values with different letters (a,b,c) in the same column are significantly different (*P* < 0.05). *WF* wheat flour, *RB* regular barley, *LBB* lean baking barley (high-amylose barley).

### Fullness and hunger

The participants’ self-evaluated hunger, satiety, fullness, desire to eat and expected prospective consumption was assessed after each of the three test meals. After 1 h we found a small reduction in hunger after LBB by 5.3%, 95% CI: 0.5, 10.2; (*P* = 0.033), compared with WF. However, no other differences were found after 1 h on the other parameters (*P* > 0.05). We found no difference after 2 h or 4 h on any of the parameters (*P* < 0.05, all).

## Discussion

This cross-over study examined 4-h postprandial glucose responses to high-amylose barley bread (LBB) versus regular barley (RB) and wheat bread (WF) in participants with and without T2D. In the T2D group, LBB reduced glucose 4 h iAUC by 41% vs. WF and 39% vs. RB. In non-T2D participants, reductions were 28% and 32%, respectively. These findings align with prior meta-analyses and highlight the potential of high-amylose starch as part of a healthy diet [15].

There is consensus that diets low in glycemic load are important for the prevention and management of diabetes and coronary heart disease, and probably obesity, particularly of relevance for individuals with insulin resistance [1, 2, 16]. Furthermore, it has been found that postprandial blood glucose is a stronger predictor of cardiovascular events than fasting blood glucose in T2D mellitus, particularly in women [17].

The reduction in glucose after LBB was simultaneously with a lowering of postprandial insulin. This lowering of postprandial insulin is closely associated with the reduction in glucose [18]. This is of great importance since hyperinsulinemia can precede and cause obesity and insulin resistance [19]. Furthermore, it has been underlined that interventions that normalise/reduce plasma insulin concentrations might play a key role in the prevention and treatment of age-related decline, obesity, T2D, CVD and cancer [2, 20, 21].

The relatively short interruption of antidiabetic treatment may have contributed to higher postprandial insulin levels in non-T2D participants compared to those with T2D. Nonetheless, we consider that the crossover study design sufficiently mitigates this potential bias and that it did not affect the observed differences in the effect of the bread types.

In addition to reduced glucose and insulin, we found a reduction in postprandial GIP after LBB compared to both RB and WF regardless of having T2D or not, and a reduction in GLP-1 after LBB compared to RB and WF in the T2D group. These findings were consistent with our previous findings with genetically modified high-amylose barley [10]. This is in line with our glucose results since higher plasma glucose concentrations are associated with greater relative insulin stimulation by GIP and GLP-1 [22]. Furthermore, the contribution of GIP to mediating the incretin effect after oral glucose in healthy human subjects is greater than that of GLP-1 [22]. This might explain why we found reduction in postprandial GIP but not in GLP-1 in the non-T2D group. Previous studies have rather consistently shown that glucose tolerance and insulin sensitivity are improved with reduced or absent GIP receptor signalling [22]. Typically, fasting GLP-1 levels are higher in individuals without T2D compared to those with T2D [23]. In our study, we found the opposite. Both DPP4 inhibitors and metformin may increase GLP-1 concentrations, and we cannot rule out that a one-day pause in antidiabetic medication may have been too short.

Nevertheless, other studies also reported higher GLP-1 levels in individuals with T2D compared to those without [24]. It has been suggested that an increase may reflect a compensatory adaptive response to elevated insulin resistance. In this study we did not measure insulin resistance.

We found no overall differences in postprandial satiety evaluations between bread types. This in in line with previous meta-analyses evaluating the effects of amylose content on postprandial subjective satiety score in healthy subjects, where no association was found between satiety and amylose content [15].

Flour from LBB has an amylose content at 47.5%, whereas flour from RB has an amylose content at 30.6% [12]. The relations between amylose content and starch digestibility is complex [25]. However, earlier studies covering the full range of amylose content in barley (0% to 100%) generally show that higher amylose content is correlated with a greater fraction of in vitro undigestible starch [26]. In a previous study using a GM method to increase amylose content in barley, the amount of resistant starch increased significantly [9, 10].

Our perception analyses of the breads showed that wheat bread was preferred over both barley breads. However, the evaluation of taste did not differ between barley breads in relation to amylose content. Taste preferences influencing food choice vary among individuals, depending on many factors such as culture, learning experiences, and genetics [27]. Given that barley is not commonly used for bread, this might to some extent explain why the taste of wheat was preferred. However, for future commercial potential, it is of high priority to work on improving the taste perception of LBB breads.

In general, it is of great importance to focus on development strategies for transformation of the agricultural sector. Modern agricultural practices, focusing on high-yielding and input-dependent monoculture cash crops, have been linked to both greenhouse gas emissions and loss of biodiversity which is of significant concern for governing unions [28, 29]. In this development process, we find it highly relevant to take the effects of crops on human metabolism into evaluation in addition to biodiversity and greenhouse gas emission. Therefore, it is of great interest to study the long-term effects of high-amylose barley in future studies.

Our findings with significantly reduced postprandial glucose following consumption of breads based on high-amylose barley (LBB), compared to wheat and regular barley, in participants with T2D as well as in participants without T2D, lead us to conclude, that that traditionally bred high-amylose barley bread may have future potential as a functional food in prevention and management of T2D.

## Data Availability

The data are available from the corresponding author upon reasonable request.
